# Functional characterization and genomic studies of a novel murine submandibular gland epithelial cell line

**DOI:** 10.1371/journal.pone.0192775

**Published:** 2018-02-20

**Authors:** Sangwon Min, Eun-Ah Christine Song, Akinsola Oyelakin, Christian Gluck, Kirsten Smalley, Rose-Anne Romano

**Affiliations:** 1 Department of Oral Biology, School of Dental Medicine, State University of New York at Buffalo, Buffalo, New York, United States of America; 2 Department of Biochemistry, Jacobs School of Medicine and Biomedical Sciences, State University of New York at Buffalo, Buffalo, New York, United States of America; University of Kentucky, UNITED STATES

## Abstract

A better understanding of the normal and diseased biology of salivary glands (SG) has been hampered, in part, due to difficulties in cultivating and maintaining salivary epithelial cells. Towards this end, we have generated a mouse salivary gland epithelial cell (mSGc) culture system that is well-suited for the molecular characterization of SG cells and their differentiation program. We demonstrate that mSGc can be maintained for multiple passages without a loss of proliferation potential, readily form 3D-spheroids and importantly express a panel of well-established salivary gland epithelial cell markers. Moreover, mSGc 3D-spheroids also exhibit functional maturation as evident by robust agonist-induced intracellular calcium signaling. Finally, transcriptomic characterization of mSGc by RNA-seq and hierarchical clustering analysis with adult organ RNA-seq datasets reveal that mSGc retain most of the molecular attributes of adult mouse salivary gland. This well-characterized mouse salivary gland cell line will fill a critical void in the field by offering a valuable resource to examine various mechanistic aspects of mouse salivary gland biology.

## Introduction

Salivary glands (SG) are exocrine glands that secrete saliva, which provides lubrication necessary for proper speech, mastication, and food tasting and hence is of critical importance for oral health. Loss of saliva secretion due to impaired acinar cell function is commonly associated with autoimmune diseases such as Sjögren’s Syndrome, from γ-irradiation therapy used in patients with oral cancers, and developmental disorders[1–3]. Patients suffering from hyposalivation exhibit difficulty in speaking, swallowing and mastication, which can reduce the quality of life. Current treatment options and targeted therapies for hyposalivation are limited to medications and the use of artificial saliva, however these options fail to provide permanent relief for patients[4]. Therefore, the generation of salivary gland specific tools and resources aimed at both a better understanding of the basic physiological and biological mechanisms important for salivary gland biology and restoring salivary gland function are important.

Over the last several years a major area of research emphasis has been aimed at restoring salivary gland function by stem/progenitor cell-based therapies and tissue engineering approaches. Indeed, using a variety of *in vitro* cell culture based strategies, numerous studies have demonstrated that human and rodent stem/progenitor cells are able to rescue radiation-induced hyposalivation in mouse models[5–9]. While such studies have shown promise, the lack of a well-defined salivary gland stem cell marker and the inherent difficulties in cultivating and maintaining SG cells have hampered progress. Although both rat and human derived cell lines have been widely used to study various aspects of salivary gland biology, often they were either derived from human tumors or immortalized using viral or recombinant DNA vectors[10–14]. Unfortunately, these strategies typically lead to phenotypic properties and molecular attributes that are distinct from normal salivary gland physiological states.

In light of these challenges, we have generated a spontaneously immortalized salivary gland epithelial cell line established from mouse submandibular glands. We show that the mouse submandibular salivary gland cell line (mSGc) can been maintained for over 100 passages without any appreciable loss of proliferation potential. Importantly, mSGc readily form 3D-spheroids and express a panel of well-established salivary gland epithelial cell markers. Moreover, we find that the 3D-spheroids exhibit secretory function as evident by agonist-induced intracellular calcium signaling. Finally, global transcriptomic characterization of mSGc and hierarchical clustering analysis reveal that the mSGc retain most of the molecular attributes of adult mouse SG. In sum, the well-characterized mSGc as described in this report adds a new toolkit in better understanding SG function.

## Materials and methods

### Animal experiments

All animal experiments were performed in compliance with Roswell Park Cancer Institute (RPCI) and the State University of New York at Buffalo, Institutional Animal Care and Use Committee (IACUC) regulations. All procedures were approved by RPCI and the State University of New York at Buffalo IACUC.

### Tissue preparation and cell culture

Twelve week old C57BL/6 male mice obtained from The Jackson Laboratory were euthanized by CO_2_ inhalation. Single cell suspensions were generated from excised submandibular glands according to Pringle *et al*.[15]. Briefly, glands were finely minced in dispersion buffer consisting of 1% (w/v) bovine serum albumin in Hank’s balanced salt solution followed by enzymatic digestion with collagenase type II (23mg/ml), hyaluronidase (40mg/ml) and CaCl_2_ (50mM) at 37°C in a shaker (125 rpm) for 20min. After 20min., digested cells were mixed vigorously and then shaken for an additional 20min. at 125 rpm. Cells were centrifuged at 400xg for 8min. and re-suspended in dispersion buffer containing enzymes and shaken for 20min. followed by vigorously mixing and shaking for an additional 20min. Cells were then centrifuged at 400xg for 8min. and washed in enzyme free dispersion buffer (1% (w/v) bovine serum albumin in Hank’s balanced salt solution) and filtered through 40μm pore filters. Cells were then grown in serum free media (SFM) consisting of Dulbecco’s Modified Eagle’s Medium (DMEM): Ham’s F12 and penicillin (100 U/ml), streptomycin (100μg/ml), human epidermal growth factor (20ng/ml), human fibroblast growth factor (20ng/ml), N2 supplement (1%), insulin (10μg/ml) and dexamethasone (1μM) and incubated at 37°C in 5% CO_2_. After 5–10 days, cell aggregates (salispheres) spontaneously adhered to the non-coated plastic tissue culture flask (Greiner Bio-One, Germany, catalog no. 658175). Once cells reached semi-confluency, cells were trypsinized using TrypLE Express (Gibco) and sub-cultured on non-coated plastic tissue culture flasks. mSGc were subsequently maintained as monolayers in either SFM or serum containing media (SCM) consisting of Dulbecco’s Modified Eagle’s Medium (DMEM):Ham’s F12 containing 2.5% Fetal Bovine Serum (FBS) (Life Technologies) and the following supplements: 0.1μM retinoic acid, 80ng/ml human epidermal growth factor, 2nM triiodothyronine, 5mM glutamax, 0.4μg/ml hydrocortisone, 5μg/ml insulin, 5μg/ml transferrin, 5ng/ml sodium selenite and 0.1mg/ml normocin.

### 3D matrigel spheroid cultures

One hundred microliters of matrigel (BD Biosciences) (2:1 matrigel:media) was allowed to solidify in a 37°C incubator for 1 hour in 8-well chambers mounted on No. 1.5 German borosilicate coverglass (Nalge Nunc International, Naperville, IL). Upon solidification, 3,000 cells/well were seeded on the matrigel in media, as indicated. 3D-spheroids were grown at 37°C in 5% CO_2_. To generate single cell suspensions from 3D-spheroids, spheres were isolated by removing media and adding 0.5ml of Dispase (1mg/ml) directly to the matrigel followed by incubation for 1 hour at 37°C in 5% CO_2_ to digest the matrigel. Spheres were washed in PBS and centrifuged at 1200rpm for 5min. Spheres were dissociated with TrypLE Express (Gibco) and passed through a 40μm filter to remove clumps and obtain single cell suspensions. Cells were then plated and grown as monolayers in either SFM or SCM.

### Growth analysis

Cells were cultured in 2ml of serum free media (SFM) or serum containing media (SCM) on non-coated plastic 6 well plates (Falcon, Corning, NY, catalog no. 353224) at a density of 250 cells/well (27 cells/cm^2^) for growth analysis. Images were taken every 24 hours for 5 days from at least 5 different fields of view using a phase contrast microscope (Nikon, Melville, NY). The number of cells per colony was calculated using Image J (NIH, Bethesda, MD). *n* = 5.

### Clonogenic assay/colony formation assay

Cells were seeded at a density of 250 cells/ well (27 cells/cm^2^) on non-coated plastic 6 well plates for five days and fixed with 4% paraformaldehyde (PFA). Fixed colonies were stained with 2% Rhodamine B in distilled water (Sigma catalog no. R6626, St. Louis, MO) and quantified. Only colonies which consisted of at least 50 cells were selected for scoring. The number of colonies per well was quantified using Image J (NIH, Bethesda, MD). The average number of colonies formed per well for each passage was calculated by adding the total number of colonies divided by the number of replicates. *n* = 4.

### Short tandem repeat (STR) analysis

IDEXX Bioresearch (Columbia, MO) performed cell line authentication by STR DNA profiling and confirmed the cells were of mouse origin and of the C57BL/6 strain.

### Spectral karyotyping

Spectral Karyotyping (SKY) analysis was performed on 20 metaphases of mSGc by the SKY/FISH facility at RPCI (Buffalo, NY).

### Immunofluorescence and confocal microscopy

Spheres were harvested by removing media and adding 0.5ml of Dispase (1mg/ml) directly to the matrigel and incubating for 1 hour at 37°C in 5% CO_2_ to digest the matrigel. Spheres were washed in Phosphate Buffered Saline (PBS) and centrifuged at 1200rpm for 5 min. Pelleted spheres were re-suspended and processed in HistoGel (Thermo Scientific) for paraffin embedding as per manufacturer’s instructions. Slides of sectioned spheres were de-paraffinized and rehydrated through a graded alcohol series. Antigen retrieval was performed by boiling slides in a microwave for 20 minutes in antigen retrieval solution (10 mM sodium citrate, 0.05% Tween-20, pH 6.0). Slides were allowed to cool for 20 min. and then rinsed briefly in PBS, circled with a PAP pen and then blocked in 20% Normal Goat Serum, 0.1% TritonX-100 in PBS for 1 hour. The primary antibodies used at the indicated dilutions include p63 (1∶25; Biocare Medical, 4A4), p63-α (1:25, Cell Signaling), K14 (1:100)[16], K5 (1:100, gift from Dr. Julie Segre NIH), K8 (1:100; Fitzgerald, MA), Nkcc1 (1:50; Santa Cruz, sc-21545), Amy1 (1:50; One World Lab), and Sma (1:200; Sigma, 1A4). When staining with mouse monoclonal antibodies MOM Basic kit (Vector Labs) was used according to manufacturer instructions. Immunofluorescence of mSGc grown as monolayers on non-coated microscope cover glasses (Fisher Scientific, catalog no.12-546) was performed as previously described[17]. Slides were mounted using Vectashield Mounting Medium with DAPI (Vector Labs) and imaged using Leica confocal microscopy.

### Cell heterogeneity analysis

mSGc cells were plated on non-coated microscope cover glasses and stained with indicated antibodies. Percentage of positive cells was calculated by counting the number of immune-positive cells and dividing by the total number of nuclei. A total of 15 fields of view were used for each quantification analysis. Each experiment was performed in triplicate. *n = 3*.

### Quantitative RT-PCR

Total RNA from mSGc grown as monolayers on non-coated plastic flasks was isolated and purified using TRIzol (Invitrogen) according to established protocols. Total RNA from spheres grown on matrigel was isolated by removing media and adding 0.5ml of Dispase (1mg/ml) directly to the matrigel and then incubated for 1 hour at 37°C in 5% CO_2_ to digest the matrigel. Spheres were washed in PBS and centrifuged at 1200rpm for 5 min. Total RNA for pelleted spheres was isolated and purified using TRIzol. Two micrograms of total RNA from mSGc grown in culture or spheres was reverse transcribed using the iScript cDNA Synthesis Kit (Bio-Rad, Hercules, CA) according to the manufacturer’s instructions. Quantitative real-time RT-PCR was performed on a CFX96 Touch Real-Time PCR machine using iQ SYBR Green Supermix (Bio-Rad). All quantitative real-time RT-PCR assays were performed in triplicate in at least three independent experiments. Relative expression values of each target gene were normalized to hypoxanthine guanine phosphoribosyltransferase (Hprt) expression. A list of the primers is provided in S3 Table.

### Intracellular calcium measurements

Relative intracellular-free Calcium (Ca^2+^) concentration was visualized in spheres grown on matrigel as described above. Cultured spheres were preloaded with 5μM Fluo-2AM (Teflabs, Austin, TX), a Ca^2+^ sensitive fluorescent dye, for 60 min. at 37°C and washed with PBS. Calcium release was measured using a Zeiss Axio Observer Z1 (Zeiss, Oberkochen, Germany) upon stimulation with 100μM carbachol (Sigma, St. Louis, MO) or 100μM ATP at room temperature. Fluo-2AM fluorescence images were captured in 10 second intervals for 10 min. using a Zeiss Axio Cam MrM camera (Zeiss, Oberkochen, Germany) and plotted using average pixel intensity values.

### RNA sequencing analysis

Isolated RNA samples were sequenced to high depth using an Illumina HiSeq platform. Sequence reads were mapped to the reference genome sequence of *Mus musculus* (mm9 build) using Tophat2[18] and transcript read counts were calculated as transcripts per million (TPM). All analyses were performed as previously described[19]. Figures were made using R v3.2.3. The heatmap program from the NMF[20] v0.23.3 R-package was used to generate all heatmaps. Counts data from the Mouse ENCODE dataset were imported and normalized as one matrix as previously described[19]. The NIH3T3 fibroblast data (https://www.encodeproject.org/experiments/ENCSR000CLW/)), and the mouse B lymphocyte, A20 data (https://www.encodeproject.org/experiments/ENCSR000CLV/) were imported with the Immortalized Mouse Oral Keratinocytes (IMOK) dataset, and combined with the counts from the mSGc dataset. The resulting matrix was normalized as one dataset and analyzed for differential gene expression using DESeq2[21] v1.12.4 R-package. Heatmaps of genes enriched in mSGc compared to the NIH3T3, A20 and IMOK cells are based on pairwise Pearson correlation of expression data with average linkage as distance measure. The correlation plot showing hierarchical clustering of mSGc and mouse tissues was generated using average linkage as distance measure on the TPM values of the top 1500 genes with the highest median absolute deviation. The heatmap showing the common genes expressed in mSGc, salivary gland, and pancreas was generated by performing pairwise Pearson Correlation with complete linkage on the log2 transformed TPM (log2(TPM+1)) values of adult mouse salivary gland gene signature (S2 Table). The resulting matrices were reordered by hierarchical clustering, after which they were transformed row-wise to report Z-scores of each gene across samples for heat-map visualization purposes.

### Statistical analysis

Results are reported as mean±S.E. (Standard Error) or mean±S.D. (Standard Deviation) of results from three or more independent experiments. Statistical comparisons were performed using unpaired two-sided Student’s *t*-test with unequal variance assumption.

## Results

### Generation of a spontaneously immortalized mouse salivary gland epithelial cell line

To generate a SG cell culture model system, cell suspensions obtained from submandibular salivary glands from 12 weeks old male C57BL/6 mice were propagated on non-coated plastic tissue culture flasks in a well-defined serum free cell culture medium[6]. After 2–3 days of growth in culture, we observed the appearance of distinct clusters of small cell aggregates, which continued their growth over the next 7–10 days and gave rise to dense clusters of 10–30 epithelial cells. The clusters, when passaged over time, lost their aggregate phenotype and gave rise to a monolayer of adherent cells with tell-tale epithelial features. We have successfully grown and maintained this mouse submandibular salivary gland derived spontaneously immortalized cell line (hereafter refer to as mSGc) as monolayers for over ~100 passages. To confirm the identity and authenticate the mSGc, short tandem repeat (STR) DNA profiling analysis and karyotyping were performed, which confirmed that the mSGc were of C57BL/6 mouse origin with no human contamination as evident from lack of human STR markers (S1 Fig). Karyotyping of early passage mSGc revealed the mouse cell line to be male and contain cell populations that have a modal chromosome number of 42 and some abnormalities to chromosomes 8 and Y (S2 Fig).

**Fig 1:**
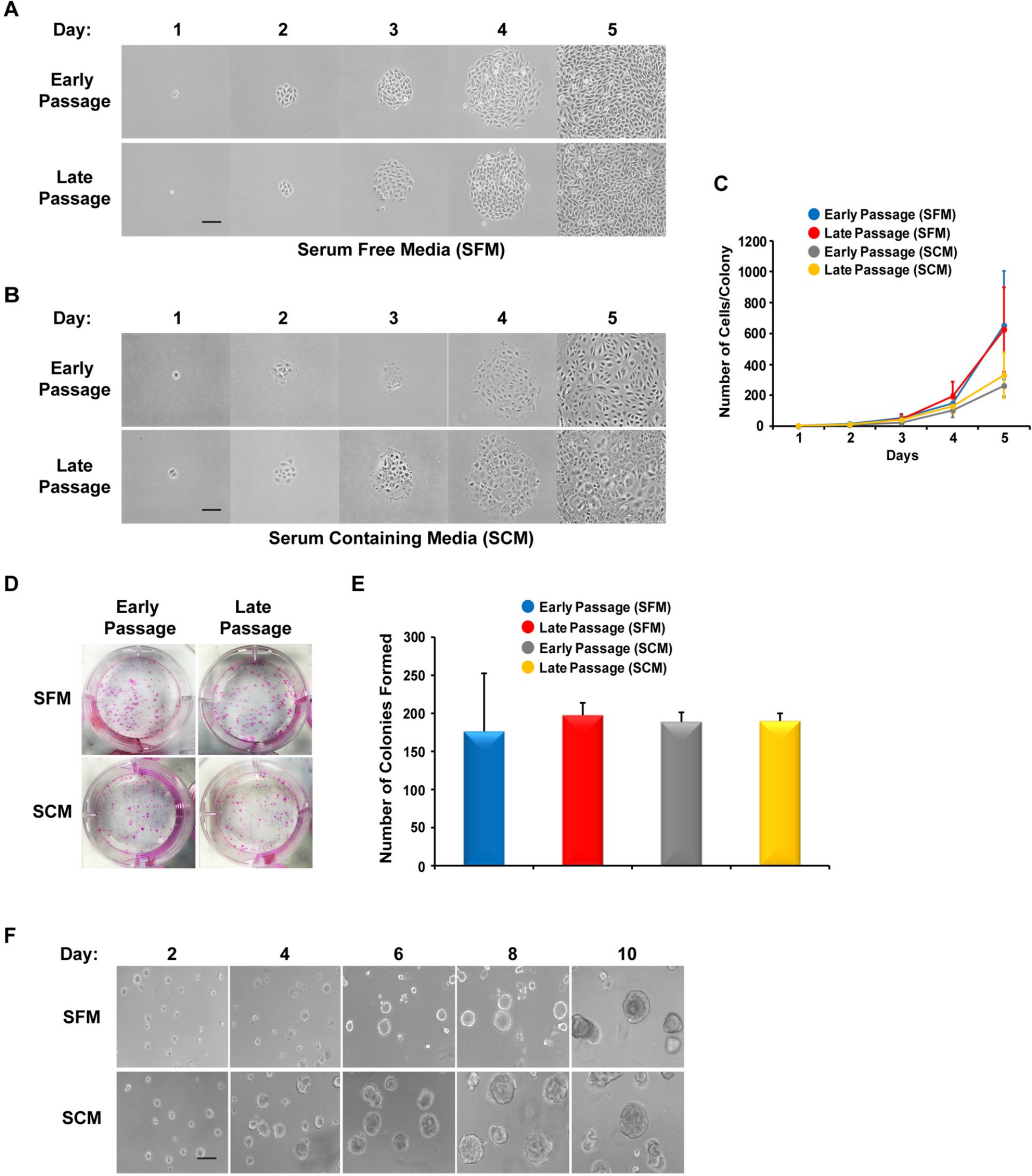
Growth analysis and 3D-spheroid formation of a spontaneously immortalized mouse salivary gland epithelial cell line (mSGc). (A-B) Early (~P20) and late passage (~P70) mSGc were seeded in serum free media (SFM) or serum containing media (SCM) as indicated, and a single-cell colony forming assay was performed. Within 5 days, large colonies were clearly visible in all cell and media conditions tested. (C) Quantification of colony forming assay performed in panel A and B. Data are represented as mean ± Standard Deviation (S.D.), *n = 5*. (D) mSGc stained with Rhodamine B revealed no differences in clonogenic potential. (E) Quantification of clonogenic results obtained in panel D above. Data are represented as mean ± Standard Deviation (S.D.), *n = 4*. (F) Late passage mSGc grown on matrigel organize into 3D-spheroid structures. Scale bar, 100μm.

To determine optimum growth conditions, we grew monolayers of mSGc in two separate cell culture medium; serum-free media (SFM)[6] or serum-containing media (SCM)[22]. Early and late passage mSGc were seeded in respective media at low density and a time-course evaluation of the colony forming ability of single mSGc was performed by counting the total number of cells per colony (Fig 1A and 1B). We found that regardless of passage number or growth media, mSGc formed colonies consisting of approximately 50 cells by day 3, which grew to 150 cells over the next 24 hours. By the fifth day in culture there appeared to be slight differences in colony forming capabilities based on the type of growth media. While mSGc grown in SFM averaged over 600 cells/colony, both early and late passage cells grown in SCM consistently gave rise to relatively smaller colonies containing roughly half the number of cells (Fig 1C). There were also slight differences in cell morphology depending on growth conditions—while cells grown in SFM displayed a polygonal morphology, cells grown in SCM appeared larger and more fusiform in appearance (Fig 1A and 1B). In addition to measuring colony forming capabilities, we investigated the ability of mSGc to generate progenies by performing clonogenic assays which revealed no differences in clonogenic capabilities based on passage number or growth conditions (Fig 1D and 1E).

**Fig 2:**
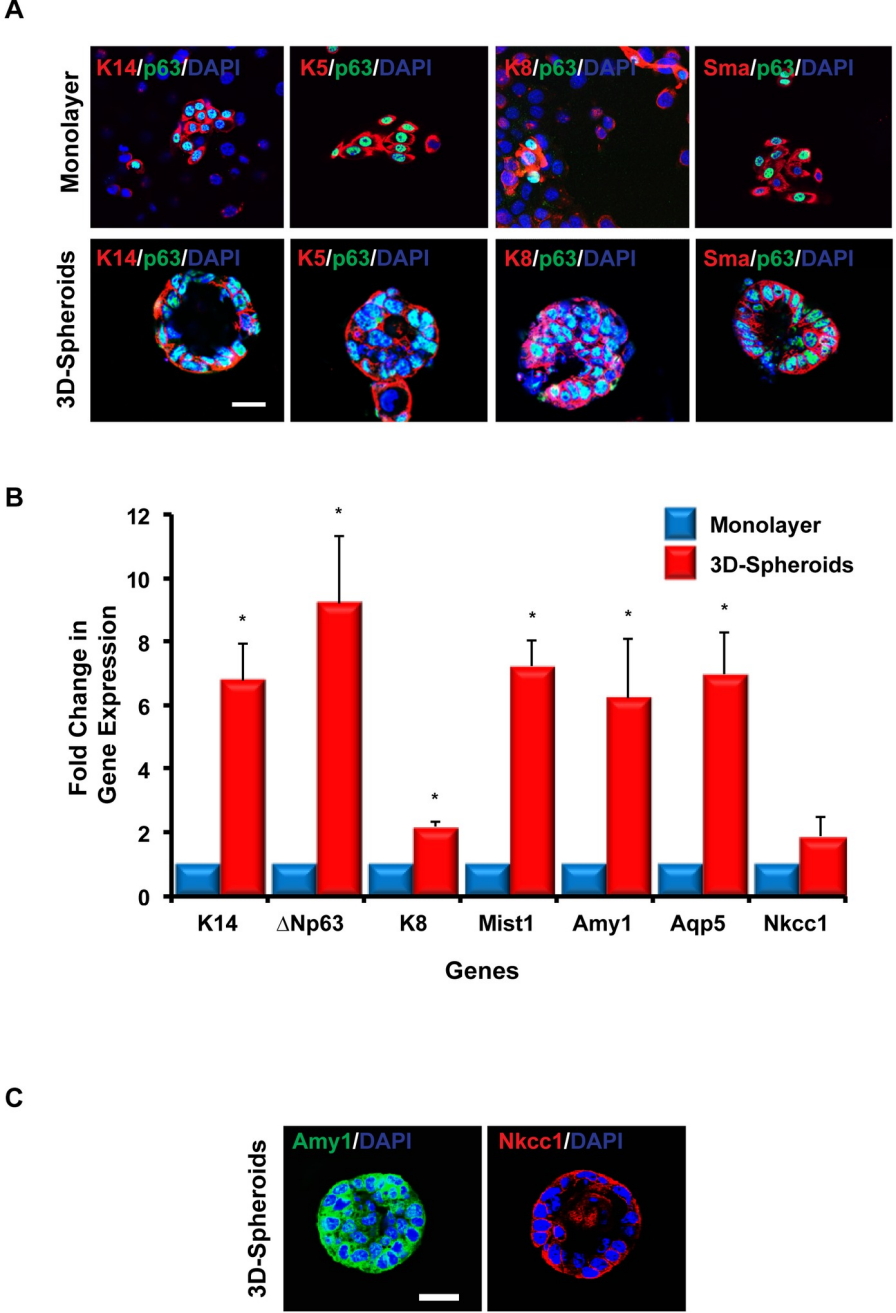
3D-spheroids express salivary gland epithelial markers. (A) Immunofluorescence staining of a sub-set of salivary gland cell markers in mSGc grown as monolayers (upper panel) and 10 day 3D-spheroids (lower panel). Scale bar 18.75 μm. (B) Quantitative RT-PCR analysis demonstrating the mRNA expression levels of a panel of salivary gland cell markers in 3D-spheroids grown for 10 days as compared to mSGc grown as monolayers. C) Immunofluorescence staining of acinar cell markers in 3D-spheroids grown for 10 days. Values were normalized to the housekeeping gene Hprt. Data are represented as the mean ±S.E. of three independent experiments. *P<0.005, Student’s *t*-test.

In parallel, we also evaluated the ability of mSGc to organize into three-dimensional (3D) spheroid structures[23]. Early and late passage mSGc were plated on a 3D matrix of matrigel and supplemented with either SFM or SCM. Within 10 days, seeded cells were capable of forming 3D sphere-like structures with well-formed lumens (Figs 1F and 2A, lower panel). To further evaluate the properties of the mSGc we tested whether cells that formed 3D spheroids, were capable of reverting and growing in their original monolayer state. Interestingly, we found that upon dispersion of 3D spheroids into single cell suspensions, mSGc were capable of growing as monolayers. Taken together, our results demonstrate that mSGc are capable of long-term expansion while maintaining their colony forming and clonogenic capabilities. Furthermore, these cells are able to organize into 3D-spheroid structures when grown in matrigel. Given the lack of any noticeable differences due to cell passage number or media conditions, all subsequent experiments were performed using mSGc maintained in SFM.

### Mouse salivary gland epithelial cells express various salivary gland cell markers

Having established an immortalized mouse salivary gland cell line and demonstrated the ability of these cells to form 3D-spheroid structures when grown in matrigel, we next sought to confirm the expression status of a panel of well-established salivary gland epithelial markers in mSGc grown as both monolayers and 3D-spheroids. Keratin 5 (K5) and K14 are epithelial cell markers that are typically expressed in both the basal cells of the ducts and the myoepithelial cells of the adult salivary gland[24–26]. Immunofluorescence analysis of K5 and K14 expression revealed robust expression of these keratins in mSGc grown as both monolayers and 3D-spheroids (Fig 2A). We also observed robust protein expression levels of the transcription factor ΔNp63 (p63), which has been shown to be highly expressed in the basal and myoepithelial cells of the salivary gland and critical for proper gland morphogenesis[27, 28], in monolayers and 3D-spheroids (Fig 2A). Keratin 8 (K8), a cytoskeletal component of the ductal cells of the salivary gland[29], was similarly expressed (Fig 2A). Interestingly, we found that both monolayers and 3D-spheroids expressed the myoepithelial cell marker alpha-smooth muscle actin (Sma) (Fig 2A)[26]. Given the observed heterogeneity of the mSGc (Fig 2A, upper panel), we next sought to better quantify the various basal, ductal, and myoepithelial enriched cell sub-populations. Interestingly, we found that approximately 45% of mSGc expressed basal cell markers (K5/14, p63), ~ 30% expressed ductal markers (K8), and ~ 48% expressed myoepithelial cell markers (K5/14, p63, Sma), further highlighting the broadly diverse cellular make-up of the mSGc, similar to the submandibular gland (S3 Fig). In parallel, using quantitative real-time polymerase chain reaction (qRT-PCR) we evaluated the expression levels of several acinar cell markers. Indeed, in addition to confirming robust mRNA expression levels of K14, ΔNp63, and K8, we found elevated mRNA expression levels of the acinar cell markers alpha amylase 1 (Amy1), aquaporin 5 (Aqp5), the transcription factor Mist1 and modest changes in expression levels of the Na^+^/K^+^/2Cl^-^ co-transporter (Nkcc1), in 3D-spheroids when compared to control mSGc grown on monolayers (Fig 2B)[22, 30]. We further confirmed protein expression levels of the acinar cell markers Amy1 and Nkcc1 in our 3D-spheroids (Fig 2C). Taken together our immunostaining and qRT-PCR data suggests that mSGc grown as monolayers and 3D-spheroids express a panel of salivary gland specific epithelial markers.

### Agonist induced responses in mSGc 3D-spheroids

**Fig 3:**
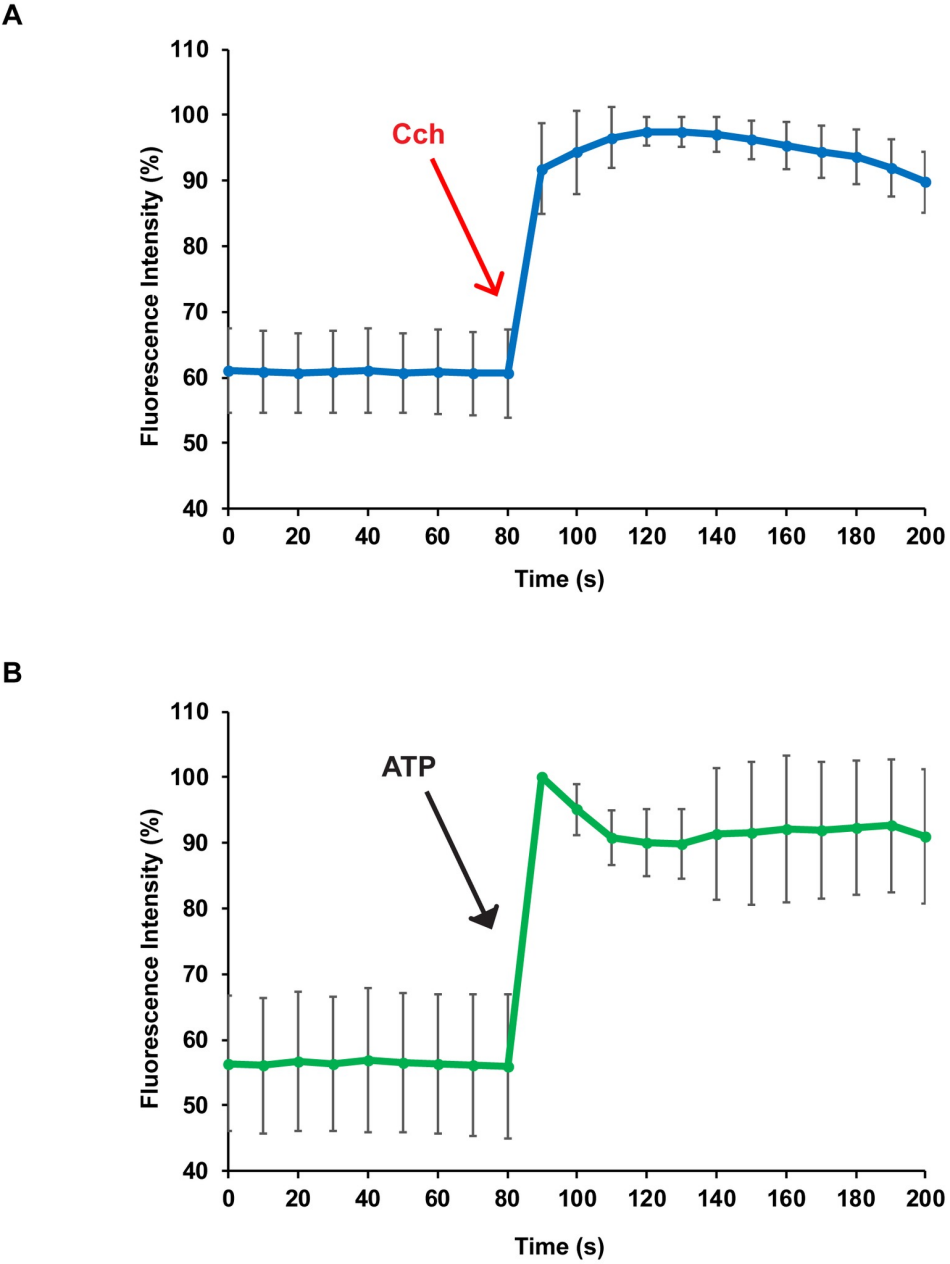
Agonist-stimulated intracellular calcium response in mSGc grown in 3D-spheroids. 3D-spheroids were stimulated with 100μM of carbachol (Cch) (A) or 100μM ATP (B) and changes in the Fluo-2AM fluorescence intensity were recorded. *n = 13* in panel A, *n = 7* in panel B. P<0.005 for both experiments, Student’s *t*-test.

The production and secretion of saliva by acinar cells of the salivary gland is a calcium (Ca^2+^) dependent process with elevated intracellular Ca^2+^ levels acting as the primary secretory signal. To determine if mSGc exhibited functional characteristics of acinar cells, we examined the ability of mSGc to respond to stimulation by various agonists by measuring changes in intracellular Ca^2+^ release. As shown in Fig 3A, when mSGc 3D-spheroids were stimulated with the muscarinic cholinergic agonist carbachol (Cch), intracellular Ca^2+^ concentrations increased immediately with a peak occurring after approximately 10 seconds. Interestingly, stimulation with the P2 nucleotide receptor agonist ATP showed similar results (Fig 3B). Taken together these data suggests that mSGc, when grown in 3D-spheroids, can response to physiological stimuli and in a characteristic fashion perform secretory cell functions.

### Defining the transcriptome of mSGc by RNA-sequencing

**Fig 4:**
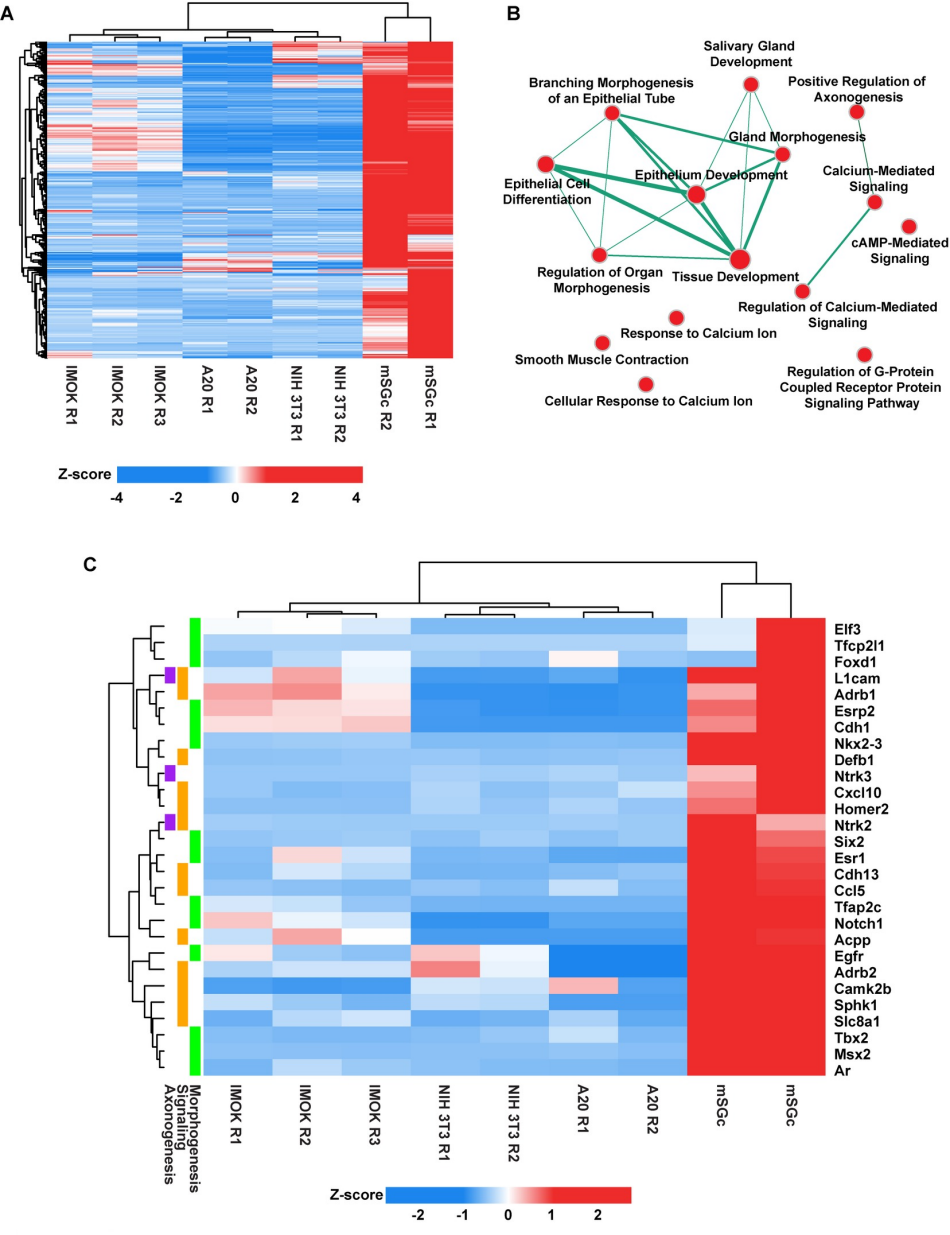
Enriched biological process networks in mSGc. (A) Heatmap visualization of genes selectively enriched in mSGc compared to A20, NIH3T3 and IMOK cells. (B) Network visualization of top enriched biological processes in mSGc as identified in panel A. Subnetworks were assembled on CytoScape[51] using Enrichment Map package[52]. Node sizes (red circles) represent the number of genes assigned to a biological process and edge width (green line) is proportional to the number of overlapping genes between two nodes. (C) Hierarchical cluster analysis of select enriched genes identified in panel A and which play a role in various biological processes as indicated by colored boxes (left panel).

To examine the global transcriptome of mSGc, we performed RNA-sequencing (RNA-seq) on cells that were grown in SFM and SCM conditions and at different passages (P2, P4, P10 and P25). In addition, the transcriptomic profile of the mSGc was also compared to similar data-sets based on RNA-seq results from cell lines of different developmental origin: NIH3T3 (fibroblasts), A20 (B lymphocytes) and Immortalized Mouse Oral Keratinocytes (IMOK) cells[31]. The close similarity of the various mSGc was quite apparent from Principal Component Analysis (PCA) as the various cell lines segregated well based on their mRNA expression profiles, as would be expected from their distinct provenance (S4 Fig). We next performed cluster analysis to identify differential gene expression patterns in mSGc, as compared to NIH3T3, A20, and IMOK cells. We specifically focused our analysis on the P10 mSGc RNA-seq dataset to make valid comparisons with IMOK cells that were analyzed at the same passage number. Indeed, we identified ~460 genes that were selectively enriched in mSGc (S1 Table) (Fig 4A). Interestingly our analysis confirmed the epithelial nature of the mSGc as we observed selective enrichment of a panel of epithelial specific genes including *Krt17*, *ΔNp63*, *Sfn*, *Krt14*, *Krt5*, *Anxa8*, *Itgb6*, and *Cdh3*. Conversely, we did not see enrichment of mesenchymal specific genes including *Mmp2*, *Twist2*, *Aspn*, *Lama2*, *Ddr2*, *Col3a1*, *Col5a2*, or B lymphocyte specific genes *Pax5*, *Cd22*, *Cd19*, *Cd40* in mSGc (S5 Fig)[32–45]. Moreover, ClueGo analysis of the ~460 mSGc-specific genes showed pathway enrichment for salivary gland development and branching morphogenesis and a number of broad biological processes related to calcium and cAMP signaling, axonogenesis and smooth muscle contraction, all of which have been shown to be required for proper salivary gland function (Fig 4B and 4C)[46–50].

**Fig 5:**
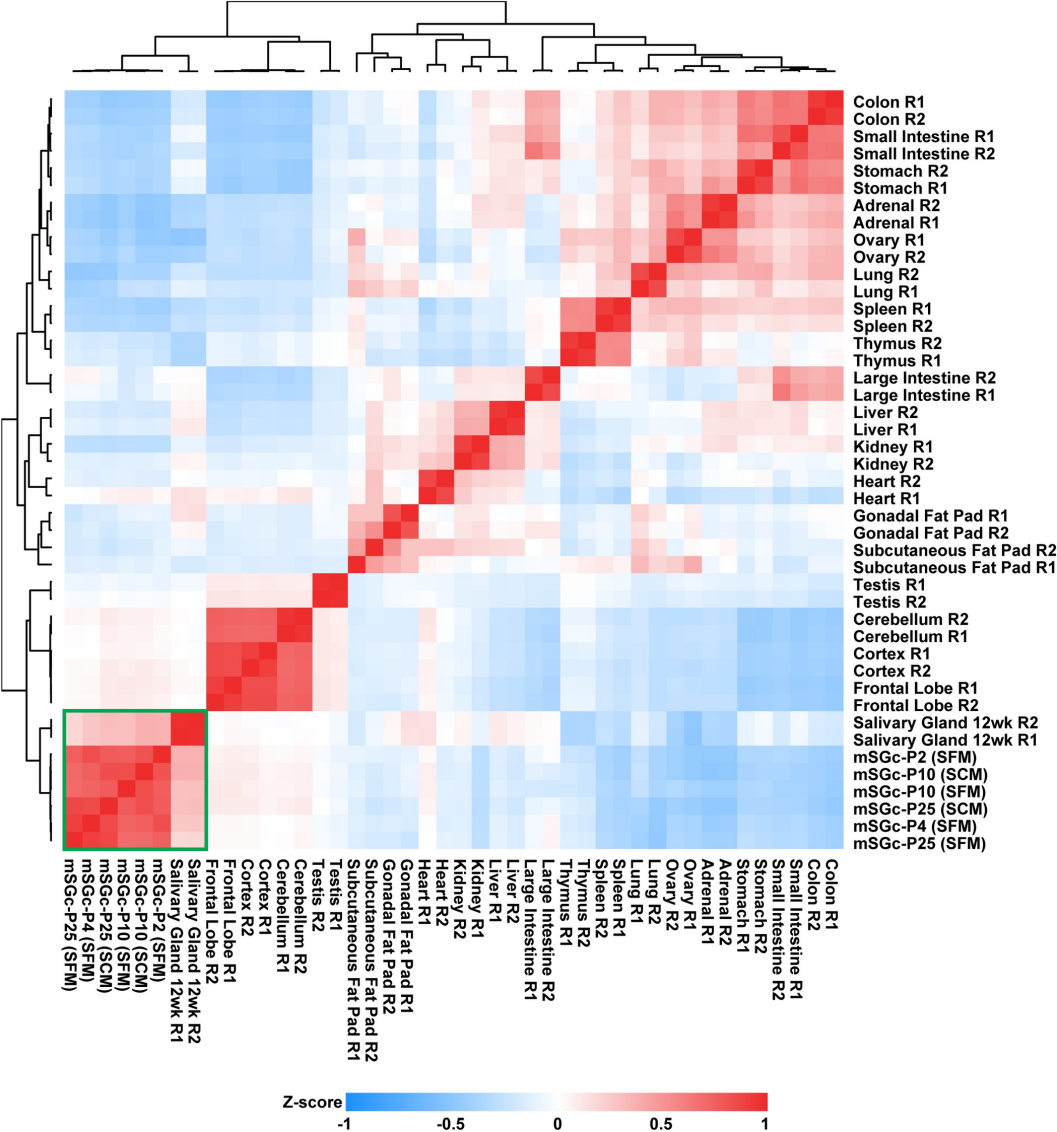
Hierarchical clustering of mSGc and mouse tissues. Transcripts per million (TPM) values from the top 1500 genes with the highest mean absolute deviation were used to cluster mSGc and adult mouse tissues (Pearson Correlation, Average Linkage). The resulting heat map demonstrates that mSGc cluster closely with the mouse salivary gland (green box).

To determine how closely the overall gene expression profile of the mSGc resembled that of the salivary gland, we next compared RNA-seq datasets of mSGc to that of mouse salivary glands generated by our laboratory and several other organs and tissues[19]. Pairwise correlation analysis revealed that the transcriptomic identity of the mSGc was most similar to the salivary gland (Fig 5, green box). We have recently identified an adult mouse salivary gland molecular gene signature of 174 genes that are specifically enriched in this gland[19]. Interestingly, of the 174 genes that made up the adult gene signature, 74 of them showed enrichment in the mSGc (S6 Fig and S2 Table). Taken together, our findings demonstrate that at the broader global transcriptomic level, the mSGc most closely resembles the salivary gland and thus can be utilized for faithful functional studies.

## Discussion

Given the valuable role mouse models have served to better understand normal biological and pathological processes in the salivary gland, the generation of a mouse salivary gland cell line that will complement mouse transgenic and knockout studies, is important. While a recent report described the long-term culture of an immortalized submandibular salivary gland epithelial cell line, no studies evaluating the sphere forming abilities or functional characteristics of these cells were performed[53]. Here we have generated and extensively characterized a spontaneously immortalized salivary gland epithelial cell line established from the adult mouse submandibular gland (mSGc). Our results demonstrate that mSGc can be cultured in various media conditions, and maintained long-term by serial passages without a loss of proliferative, clonogenic, or sphere forming potential.

One interesting feature of the mSGc is that similar to the salivary gland, mSGc grown as monolayers express a number of salivary gland specific markers including basal (K5/K14), ductal (K8), and myoepithelial (Sma) cell markers, suggesting that these cells represent a heterogeneous population. Yet another distinguishing feature of the mSGc is their ability to organize into 3D-spheroid structures when grown in matrigel. Interestingly, we found that 3D-spheroid structures express salivary gland specific markers which show a distinct spatial organization similar to that of the salivary gland. More specifically, we observed expression of K5/K14 and Sma located towards the outer periphery of the lumen enriched spheres highlighting the ability of the mSGc derived spheres to form polarized structures. Moreover, we observed expression of the acinar cell markers Amy1, Mist1, Aqp5 and Nkcc1 which is in good agreement with the observed intracellular Ca^2+^ release upon stimulation with both the muscarinic cholinergic agonist, carbachol (Cch) and the P2 nucleotide receptor agonist, ATP, suggesting secretory function of the 3D-spheroid structures.

While determining the optimal growth conditions for mSGc, we noticed that there were no discernible differences in colony forming, clonogenic or sphere forming capabilities when grown in the presence (SCM) or absence (SFM) of serum. While there are several plausible explanations for the lack of serum-induced effects, we suspect one potential explanation is that our SCM contains relatively low levels of serum (2.5%). These levels are considerably lower than serum levels commonly used to grow and cultivate epithelial cells, which is typically 10%. Furthermore, it should be stressed that both the SFM and SCM utilized for our studies contain the growth factor EGF, which may be sufficient for mSGc survival and growth in our culture conditions. Alternatively, it is possible that the inherent nature of the immortalization process may have forced the cells to adapt to minimal and/or no growth factor requirements. Indeed, the ability of mSGc to be propagated both in the presence or absence of serum is also in good agreement with our findings in which the mSGc and primary salivary gland cells can be maintained in a defined serum free medium. These observations are not unique to the salivary gland cells described in this manuscript, as defined serum free medium is commonly used to grow primary oral and epidermal keratinocytes[54, 55]

Transcriptional profiling of mSGc at different passages and growth conditions and after comparison to other cell lines, revealed specific enrichment of genes associated with salivary gland secretion and calcium signaling, further highlighting the preservation of the tissue specific properties and functions of mSGc. Our findings from hierarchical clustering analysis reveals that mSGc grown in culture and the mouse salivary gland, share a conserved network of crucial genes and pathways and underscores the overall value and usefulness of our newly established immortalized mouse salivary cell line to better study salivary gland biology. With increased focus aimed at developing strategies to regenerate or repair salivary glands using stem/progenitor cells or using SG cells in tissue engineering approaches, there remains a need for a firm understanding of the underlying biological properties of the cell types being employed. Using a variety of molecular and biochemical approaches, we demonstrate that mSGc offer an additional resource to examine molecular and mechanistic aspects important for salivary gland development and salivary gland biology overall, with the long-term goal to treat diseases associated with salivary gland dysfunction.

## Supporting information

S1 Fig STR analysis of mSGc. Analysis demonstrates that mSGc are exclusively of C57BL/6 mouse origin. Moreover, there is no human cell contamination as evidenced by a lack of human STR markers.
(TIF)

S2 Fig Spectral karyotyping of mSGc. Spectral karyotyping (SKY analysis) of metaphases from mSGc. An inverted DAPI image depicting chromosome banding is shown in the upper panel. Lower panel demonstrates SKY karyotype after chromosome classification.
(TIF)

S3 Fig mSGc represent a heterogeneous cellular population. Quantitative RT-PCR analysis demonstrating mRNA levels of various epithelial markers in mSGc grown as monolayers. Values were normalized to the housekeeping gene Hprt. Data are represented as the mean ±S.E. of three independent experiments.
(TIF)

S4 Fig Principal Component Analysis (PCA). Projection plots show the PCA coordinates for each of the mSGc samples, NIH3T3 (fibroblasts), Immortalized Mouse Oral Keratinocytes (IMOK) and A20 (B lymphocytes) cells.
(TIF)

S5 Fig Enrichment of epithelial specific genes in mSGc. A heatmap visualization of epithelial enriched genes in mSGc cells compared to A20, NIH3T3 and IMOK cells.
(TIF)

S6 Fig Preservation of the tissue specific adult mouse salivary gland gene signature in mSGc. Hierarchical clustering of mSGc and mouse tissues using averaged TPM values of the genes that make up the adult mouse salivary gland gene signature.
(TIF)

S1 Table Genes uniquely expressed in mSGc. (XLSX)

S2 Table Common genes between mSGc and the adult mouse salivary gland gene signature. (XLSX)

S3 Table List of primers. (XLSX)
