# Supplementary figures and images for: Functional characterization and genomic studies of a novel murine submandibular gland epithelial cell line
Source: PLoS One. 2018 Feb 20;13(2):e0192775. doi: 10.1371/journal.pone.0192775 (PMC5819789; doi:10.1371/journal.pone.0192775)

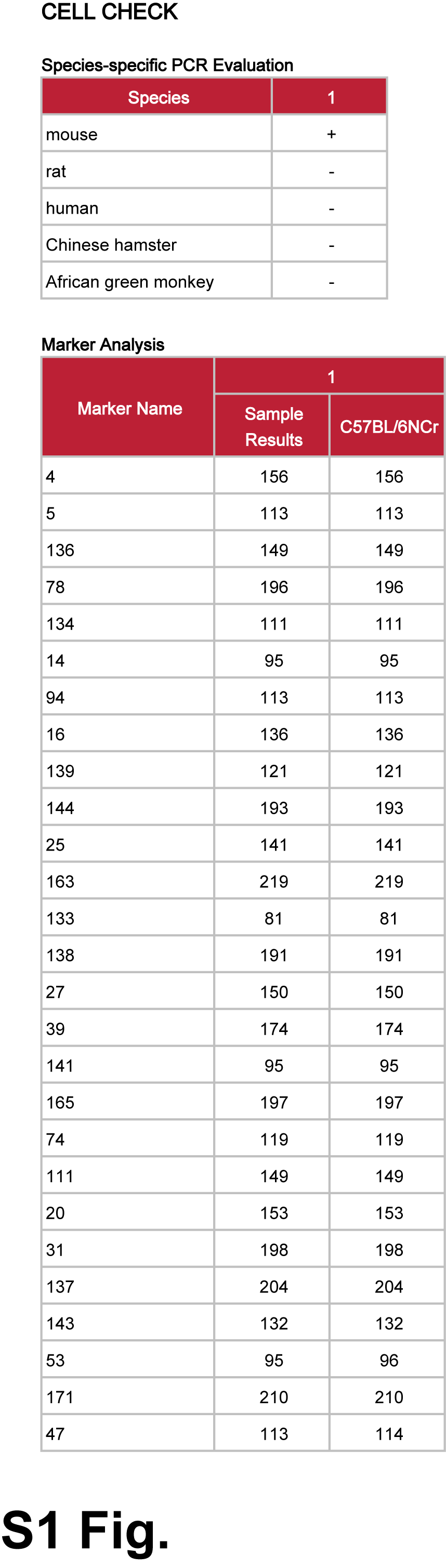

Supplement: S1 Fig — Analysis demonstrates that mSGc are exclusively of C57BL/6 mouse origin. Moreover, there is no human cell contamination as evidenced by a lack of human STR markers. (TIF) [file pone.0192775.s001.tif]

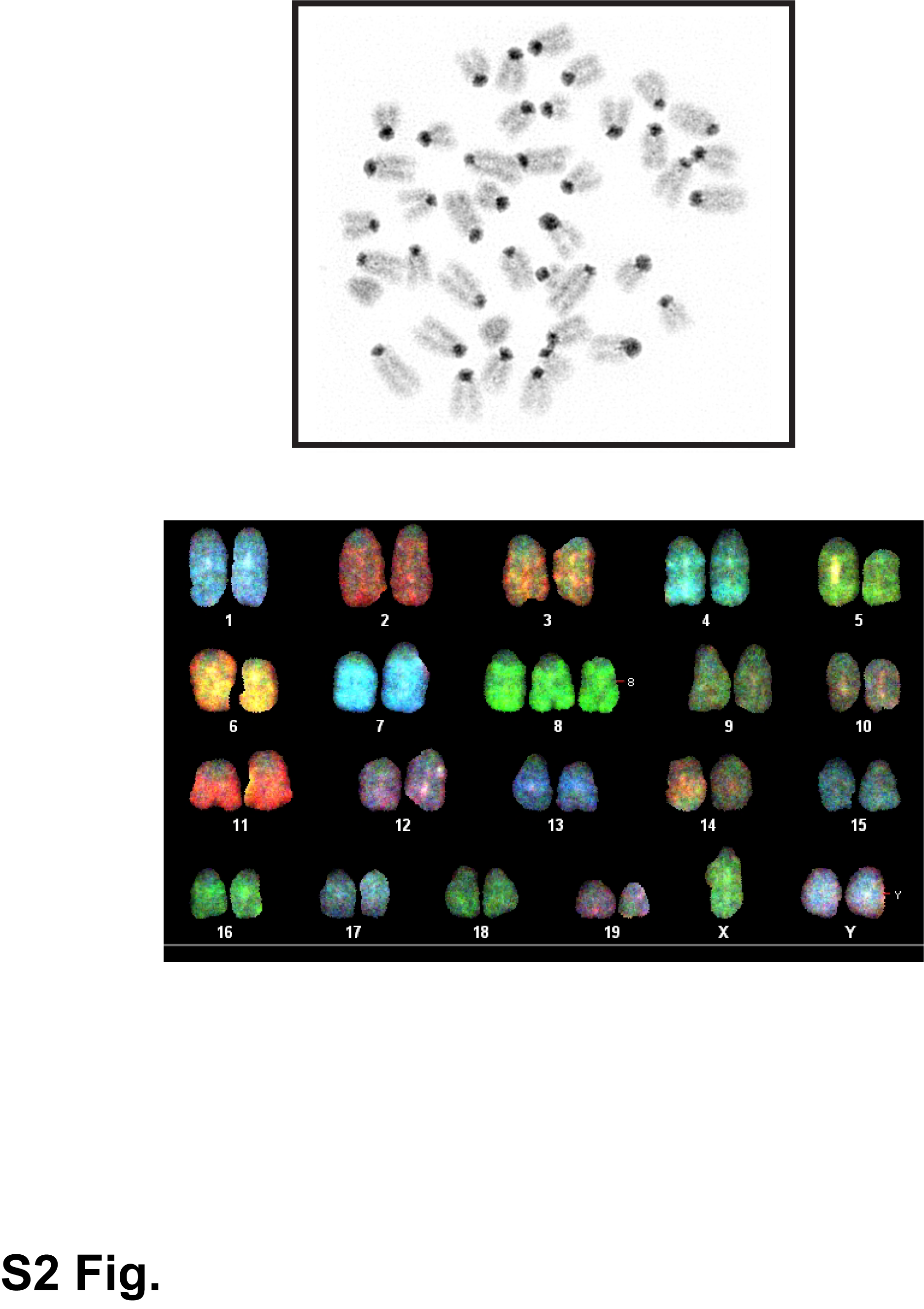

Supplement: S2 Fig — Spectral karyotyping (SKY analysis) of metaphases from mSGc. An inverted DAPI image depicting chromosome banding is shown in the upper panel. Lower panel demonstrates SKY karyotype after chromosome classification. (TIF) [file pone.0192775.s002.tif]

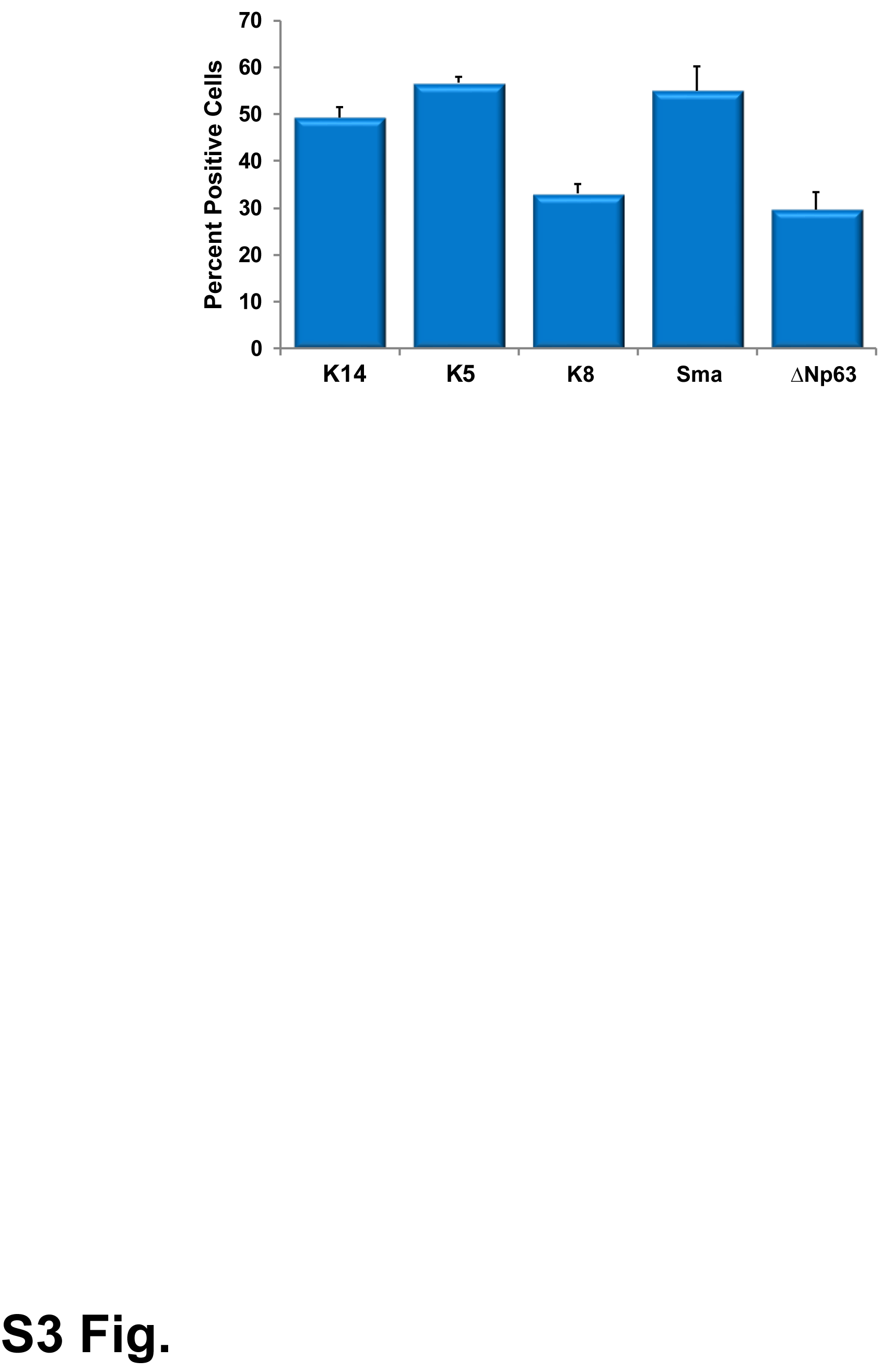

Supplement: S3 Fig — Quantitative RT-PCR analysis demonstrating mRNA levels of various epithelial markers in mSGc grown as monolayers. Values were normalized to the housekeeping gene Hprt. Data are represented as the mean ±S.E. of three independent experiments. (TIF) [file pone.0192775.s003.tif]

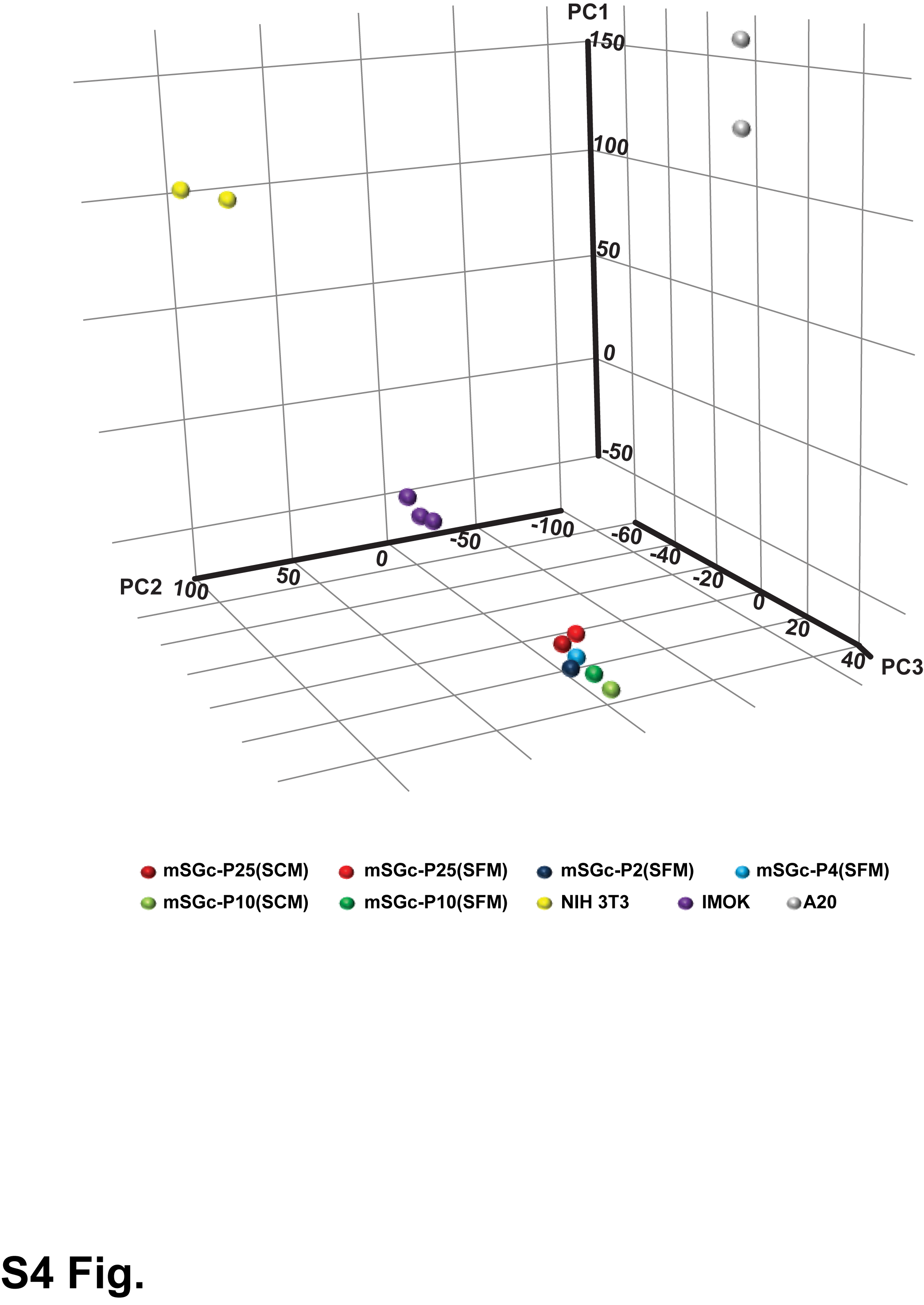

Supplement: S4 Fig — Projection plots show the PCA coordinates for each of the mSGc samples, NIH3T3 (fibroblasts), Immortalized Mouse Oral Keratinocytes (IMOK) and A20 (B lymphocytes) cells. (TIF) [file pone.0192775.s004.tif]

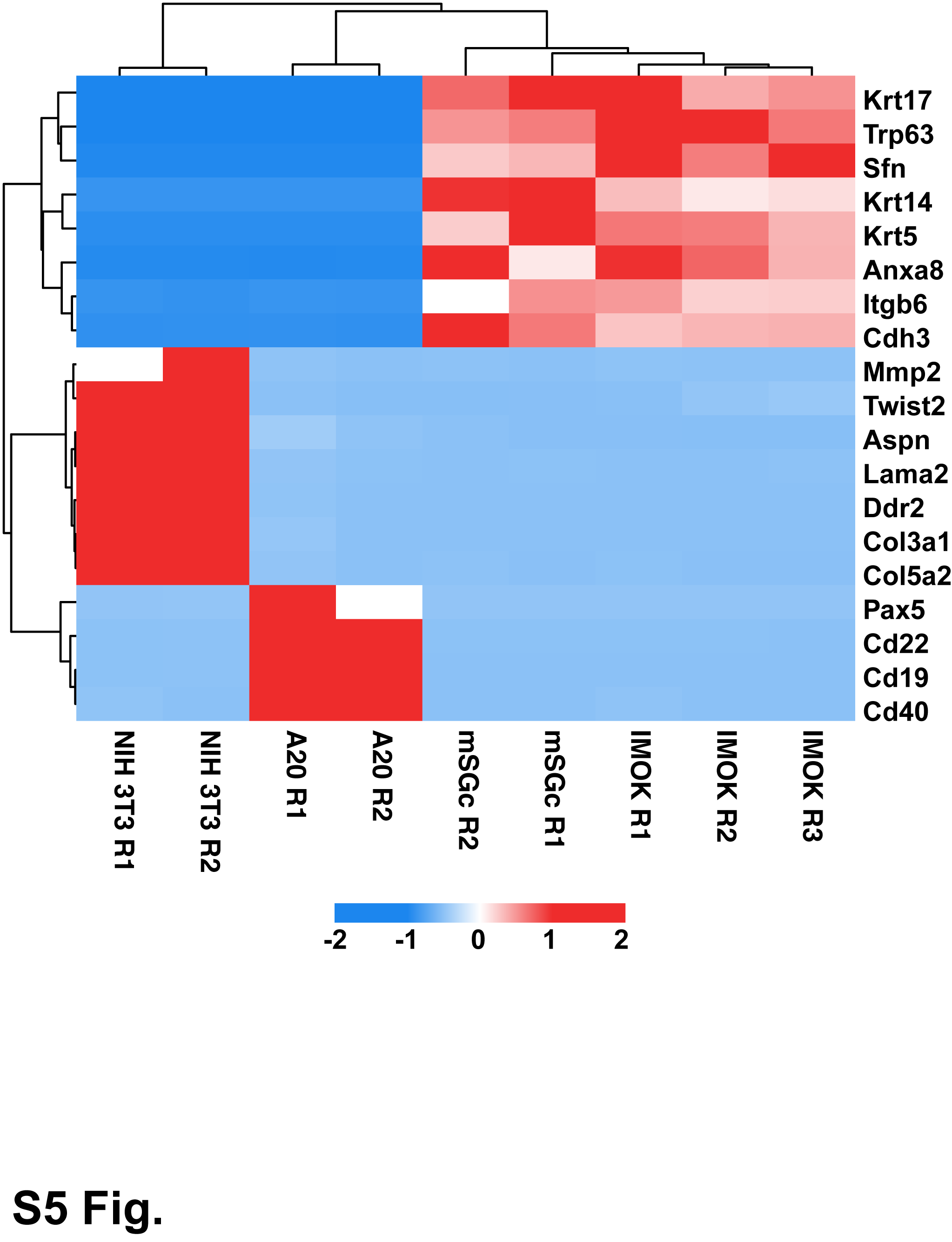

Supplement: S5 Fig — A heatmap visualization of epithelial enriched genes in mSGc cells compared to A20, NIH3T3 and IMOK cells. (TIF) [file pone.0192775.s005.tif]

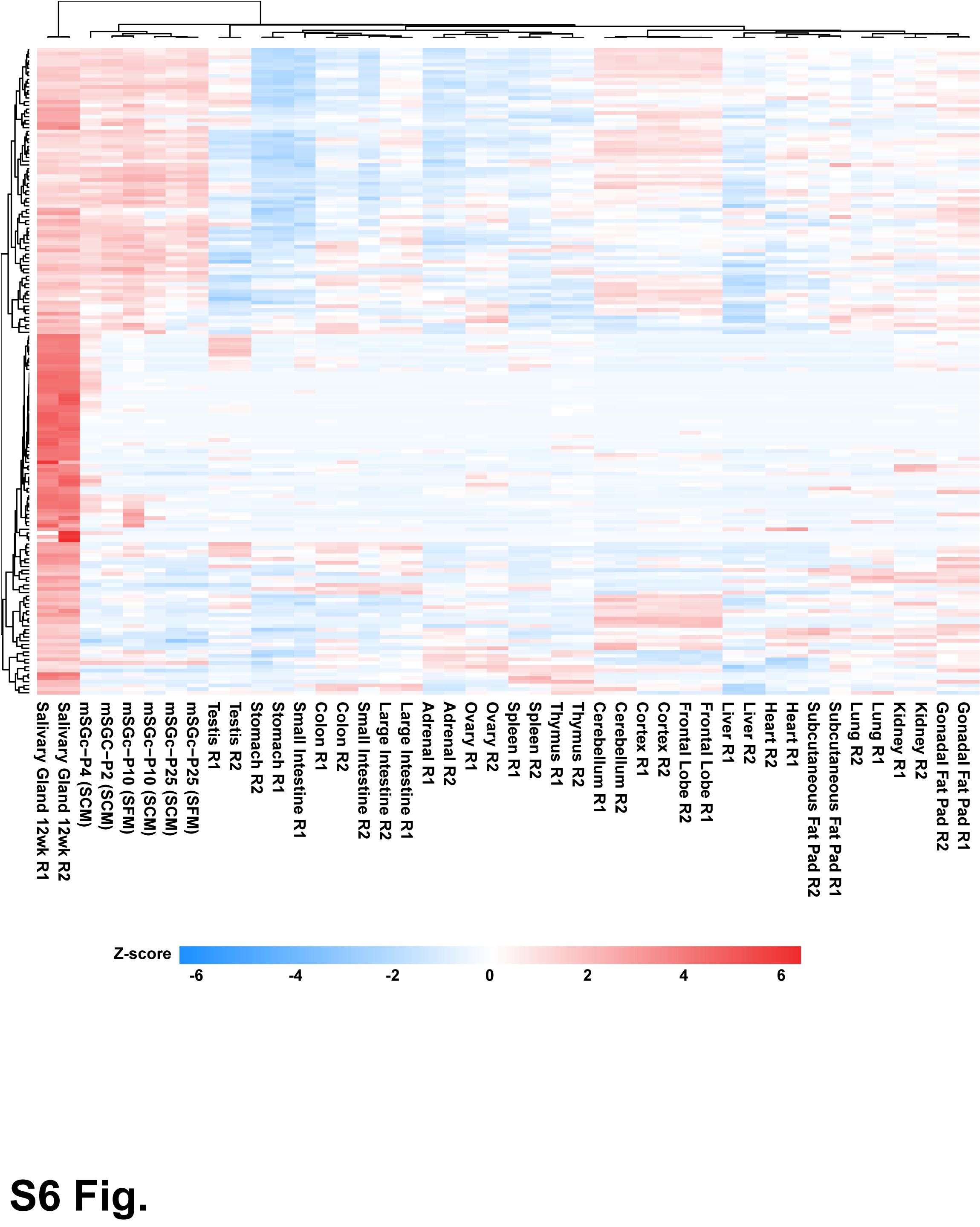

Supplement: S6 Fig — Hierarchical clustering of mSGc and mouse tissues using averaged TPM values of the genes that make up the adult mouse salivary gland gene signature. (TIF) [file pone.0192775.s006.tif]
